# Synthesis, Characterization, and Investigation of the Properties of a New Promising Poly(Azomethine) Organic Semiconductor Material

**DOI:** 10.3390/ma18071658

**Published:** 2025-04-04

**Authors:** Jihane Ismaili, Chouki Zerrouki, Najla Fourati, Stephanie Leroy-Lhez, Daniel Montplaisir, Nicolas Villandier, Rachida Zerrouki

**Affiliations:** 1 LABCiS, Faculté des Sciences et Techniques, Université de Limoges, 123 Avenue Albert Thomas, 87060 Limoges, France; jihane.ismaili@rmc-cmr.ca (J.I.); stephanie.lhez@unilim.fr (S.L.-L.); nicolas.villandier@unilim.fr (N.V.); 2 Département de Biochimie, Chimie, Physique et Science Forensique, Université du Québec à Trois-Rivières, Trois-Rivières, QC G9A 5H7, Canada; daniel.montplaisir@uqtr.ca; 3 Laboratoire SATIE, UMR 8029, CNRS, ENS Paris-Saclay, Cnam, Conservatoire National des Arts et Métiers, 75003 Paris, France; zerrouki@cnam.fr (C.Z.); fourati@cnam.fr (N.F.)

**Keywords:** organic semiconductors, chemical synthesis, physical characterizations, optical properties, semiconductor material

## Abstract

A new poly(azomethine) with improved solubility was successfully prepared by the polycondensation of terephthalaldehyde and 2,2-Bis[4-(4-aminophenoxy)phenyl]-hexafluoropropane (4-BDAF) under green chemistry conditions. This new polymer containing hexafluoroisopropylidene was compared with a polymer containing isopropylidenediphenyl to study the influence of the presence of fluorine atoms on the properties of the polymer. Both were characterized by nuclear magnetic resonance (NMR), their molecular weight was measured by gel permeation chromatography (GPC), and their morphology was studied by X-ray diffraction (XRD). The two polymers obtained were soluble in most polar aprotic solvents and even in less polar solvents, which are practical and easily accessible solvents. Their thermal properties were determined by a thermogravimetric analysis (TGA) and differential scanning calorimetry (DSC). These two new polymers showed high resistance to thermal decomposition up to 490 °C, with a glass transition temperature (Tg) of 180 °C. The photophysical properties were studied by UV/Visible absorption. The polymers were doped and then deposited on cellulose filaments, an approach that made it possible to produce self-supporting conductive composites thanks to their mechanical properties. The topography of the resulting materials was characterized at submicron scales before estimating their electronic conductivity and gap energy by diffuse reflection spectroscopy.

## 1. Introduction

Highly conjugated polymers have attracted much attention in the last few years as promising candidates for a wide variety of applications such as electronics, opto-electronics, photonics, and energy storage [1,2,3,4,5,6]. Among these polymers, poly(azomethines) containing CH=N- groups in the backbone, also called poly(imines) or Schiff-base polymers, have received increasing interest because of their isoelectronicity with poly(p-phenylene vinylene), the most electroluminescent polymer [7].

The synthesis of poly(azomethines) occurs from the polycondensation of diamines and dicarbonyl monomers. This reaction exclusively ensures the formation of azomethine without any undesired byproduct. They do not require synthetic and purification protocols as complex as those used in conventional aryl–aryl coupling protocols [8].

A wide variety of conjugated poly(azomethines) have been synthesized using different aromatic backbones and several side substituents, and their electronic and optical properties have been investigated [4,9,10,11]. However, the major drawback of high molecular weight poly(azomethines) is their limited solubility in common organic solvents. In order to improve their solubility, several modifications have been reported, such as the introduction of pendent groups (aromatic, alkoxy, or alkyl groups) onto the polymer chain and/or the incorporation of non-coplanar structural units into the main chain [12,13]. Polycondensation in a mixture of solvents or solvent/salt and the inclusion of flexible spacers between main-chain aromatic rings have also been reported [13,14,15].

Among these flexible spacers, polymers containing hexafluoroisopropylidene groups have been intensively investigated [16,17,18,19,20]. The incorporation of fluorine atoms (or fluorine-containing groups) increased solubility, thermal stability, flame retardancy, glass transition temperature, oxidation resistance, and environmental stability [16]. The use of bulky hexafluoroisopropylidene groups also allowed an increase in the free volume of polymers, improving the electrical insulation characteristics [17,19]. More recently, aromatic fluorine-containing polymers have also been investigated for applications in gas-separation membranes [19], hole-transporting materials [9], charge-transporting materials, and polymer electrolyte membrane fuel cells [21,22,23].

**Scheme 1:**
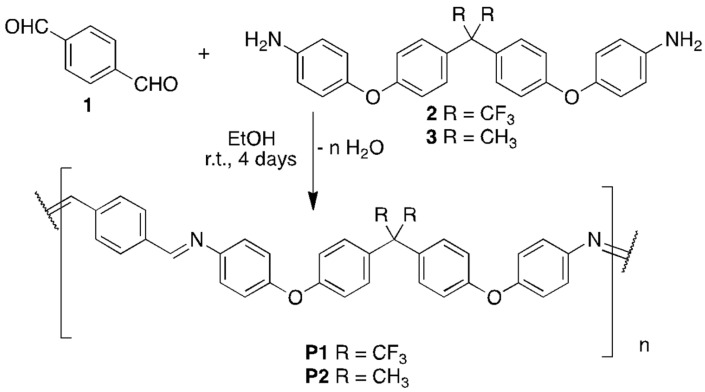
Synthetic route and chemical structure of synthesized poly(azomethines).

In this study, we report the synthesis and characterization of a new poly(azomethine), **P1** (Scheme 1), with improved solubility from the polycondensation of 2,2-Bis[4-(4-aminophenoxy)-phenyl]hexafluoropropane (4-BDAF) and terephthalaldehyde at room temperature. This new hexafluoroisopropylidene-containing polymer was compared with an isopropylidenediphenyl-containing polymer, **P2** (Scheme 1), in order to study the influence of fluorine atoms on the polymer properties. A full investigation was conducted to highlight the polymer properties, i.e., the atomic structure (nuclear magnetic resonance, or NMR), molecular weight (gel permeation chromatography, or GPC), morphology (X-ray diffraction, or XRD), thermal behavior (thermogravimetric analysis, or TGA, and differential scanning calorimetry, or DSC), and opto-physical characteristics (UV/Visible absorption).

## 2. Experimental Section

### 2.1. Materials

2,2-Bis[4-(4-aminophenoxy)phenyl]hexafluoropropane (4-BDAF; 97%), 2,2-Bis[4-(4-aminophenoxy)phenyl]propane (BAPP; 98%), terephthalaldehyde (99%), and absolute ethanol were purchased from Sigma-Aldrich ((Lyon, France)) and used without further purification. The tetrahydrofuran (THF, ≥99.7%, and purchased from Alfa Aesar (now thermo fisher scientific, Paris, France)) used for the UV/Vis measurements was of spectroscopy grade and was stored in a dark place. Reactions were monitored by thin layer chromatography (TLC) using precoated 0.2 mm silica gel 60 F254 (Merck) plates and visualized using an ultraviolet light source at 254 nm.

### 2.2. Synthesis of Polymers **P1** and **P2**

The poly(azomethines) **P1** and **P2** were synthesized by the polycondensation reaction of 1.8 g (13.42 mmol) terephthalaldehyde and 7 g (13.5 mmol) 2,2-Bis[4-(4-aminophenoxy)phenyl]-hexafluoropropane (4-BDAF) or 5.5 g (13.40 mmol) 2,2-Bis[4-(4-aminophenoxy)phenyl]-propane (BAPP) as diamino compounds in 50 ml absolute ethanol at room temperature for 4 days under stirring. The formed yellow precipitate was filtered; washed 3 times with ethanol, acetone, and diethyl ether; and then dried. Polymer **P1** was obtained with an 88.6% mass yield (7.8 g) and polymer **P2** had an 97.3% mass yield (7.1 g).

### 2.3. Instruments

^1^H NMR spectra were recorded using a Varian 200 MHz NMR spectrometer (Wissembourg, France) Chemical shifts (δ) were expressed in parts per million with Me_4_Si as an internal standard (δ = 0).

Gel permeation chromatography (GPC) was used to determine the polymers’ number-average (Mn) and weight-average (Mw) molecular weights as well as the polydispersity (Mw/Mn) via an Agilent 1200 series HPLC. Polystyrene standards were used for calibration [18]. The column used was a PLgel mixed-D (5 μm) and the detector was a refractive index detector (RID). All samples were dissolved in THF (5 mg/5 mL) for 5 min to allow complete dissolution before being filtered with a PTFE filter (0.45 μm). The column was operated at 40 °C and eluted with THF at 0.5 mL/min. The RID was maintained at 35 °C. The injected sample volume was 20 μL.

UV/Vis spectra were recorded using a Specord 210 (Analytik Jena) double-beam spectrophotometer with 10 mm quartz cells.

Thermogravimetric analyses (TGAs) were performed from 80 to 900 °C using a q500 apparatus (TA instruments) at a constant heating rate of 20 °C/min under a nitrogen flow of 50 ml/min. Samples were contained in platinum pans. Differential scanning calorimetry (DSC) measurements were made using a DSC q10 (TA instruments) operating from 0 to 300 °C under a nitrogen flow at a constant heating rate of 10 °C/min.

XRD measurements were obtained using a homemade goniometer [24,25]. The X-ray pattern was obtained using K_α2_-filtered Cu K_α1_ radiation (Si 111 crystal monochromator) and a Seifert X-ray generator at 35 kV and 35 mA. The scan range was 3°–40° and the sampling interval was 0.02°.

Conductivity measurements were obtained using a four-point collinear probe connected to a source measure unit (SMU) instrument (Tektronix model 2450).

Atomic force microscopy was performed using a Nanosurf easyScan 2 AFM in non-contact phase contrast mode and operating at a resonance frequency of approximately 200 kHz. The probes utilized were ultra-sharp silicon, featuring a half-cone angle of less than 10° at 200 nm from the apex along with a nominal curvature radius of less than 2 nm [26].

Diffuse reflectance spectroscopy (DRS) was performed using a Shimadzu 00072 spectrophotometer covering a wavelength range from 200 to 1400 nm with a step size of 0.1 nm.

## 3. Results and Discussion

### 3.1. Synthesis and Characterization

#### 3.1.1. Synthesis

Poly(azomethines) are generally synthesized in an inert atmosphere using catalysts, at elevated temperatures, and in organic solvents such as dimethylacetamide (DMAc), dimethylformamide (DMF), or tetrahydrofuran (THF) [9,27,28,29,30,31]. Hakimi et al. carried out this synthesis in ethanol (considered to be a green solvent [32]) always under reflux and using a catalyst [33,34].

In this work, we chose environmentally friendly conditions by using ethanol at room temperature and without using catalysts or strong Lewis bases (Scheme 1). The polycondensation reaction of the corresponding diamines 2 or 3 with terephthalaldehyde 1 gave mass yields of 91% and 97% for polymers **P1** and **P2**, respectively, and their chemical structure was characterized by NMR spectroscopy.

#### 3.1.2. ^1^H NMR Analysis

**Figure 1:**
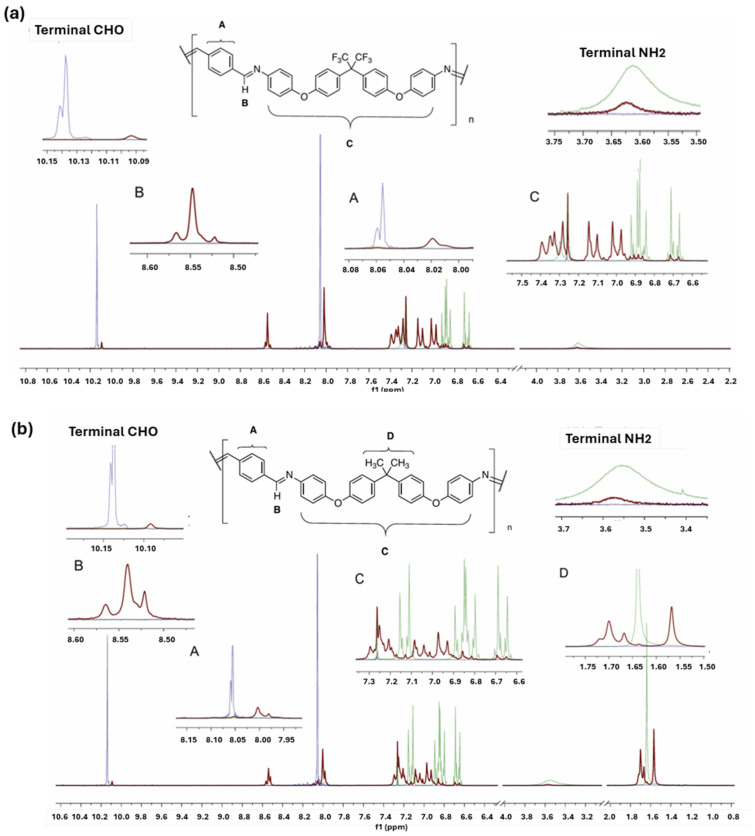
^1^H NMR spectra of (**a**) polymer **P1** in red, diamine **2** in green, and dialdehyde **1** in blue and (**b**) polymer **P2** in red, diamine **3** in green, and dialdehyde **1** in blue.

Polymers **P1**, **P2,** and their precursors **1**, **2**, and **3** were analyzed by ^1^H NMR in CDCl_3_ (Figure 1). In the figure, functional groups (or parts) of the polymer are labelled A, B or C. The corresponding parts of the spectrum are also noted in the same way. The NMR spectra showed the formation of imine groups in both polymers **P1** and **P2** by the presence of signals in the range of 8.5–8.6 ppm (Figure 1, part B) confirming the polycondensation reaction. We noticed that the aromatic protons of the polymer (Figure 1, part A) were down-field shifted with respect to the terephthalaldehyde protons because of the lower-withdrawing effect of the imine function compared with the aldehyde function. On the other hand, a significant up-field shift of the diamine’s aromatic region (Figure 1, part C) was observed in both polymers **P1** and **P2** due to a weaker donor effect of nitrogen in the imine function. Finally, we observed the presence of terminal aldehyde and amine functions at 10.1 and 3.6 ppm, respectively.

#### 3.1.3. GPC Analysis

The weight-average molecular weight (Mw) of these polymers was measured by GPC using polystyrene standards and THF as an eluent (Table 1) [18]. We noticed that, at room temperature, we obtained an oligomeric compound averaging 8 repeated units (Mw = 4903 g/mol) in the case of polymer **P1** and 5 repeated units (Mw = 2324 g/mol) in the case of polymer **P2**. Molecular distributions were within a relatively narrow range for both polymers (i.e., 1.54 and 1.17 for **P1** and **P2**, respectively; Table 1). Compared with polymer **P1**, **P2** was closer to a monodisperse distribution. It was, therefore, slightly more homogeneous in terms of the size/mass of the basic molecular unit. This difference was reflected in the appearance of more ordered domains within **P2**, giving rise to diffraction peaks visible in the XRD measurements. Regarding the thermal properties, the **P1** polymer stood out because of its non-uniform behavior in relation to dispersion.

#### 3.1.4. Solubility Behavior

One of the important factors for low-cost processing methods such as printing, stamping, and curtain-coating is the solubility behavior of the polymer, which is essential to the commercial success of organic electronics. Therefore, the solubility behavior of these new polymers was tested using various organic solvents, and the results are summarized in Table 2. Polymer solubility was qualitatively determined by the dissolution of 1 mg of the solid polymer in 1 ml organic solvent at room temperature. Both polymers were easily soluble in most polar aprotic solvents (DMAc; NMP) and even in less polar ones such as CHCl3 and THF, which are convenient and easily accessible solvents. Moreover, we noticed, in the case of polymer **P1**, better solubility behavior in a more concentrated solution (20 mg/ml) of some solvents such as CHCl3 and THF, while polymer **P2** had limited solubility at a concentration of 1 mg/ml. In addition, polymer **P1** was soluble in DMF but polymer **P2** was not. The CF3 group increases solubility, and the introduction of flexible aryl-ether linkages in the polymer backbone is generally known to have an impact on properties such as better solubility, melt-processing characteristics, and improved toughness compared with polymers without aryl–ether linkages [21,22]. Their good solubility makes them potential candidates for practical applications in spin-coating and casting processes.

#### 3.1.5. XRD Analysis

**Figure 2:**
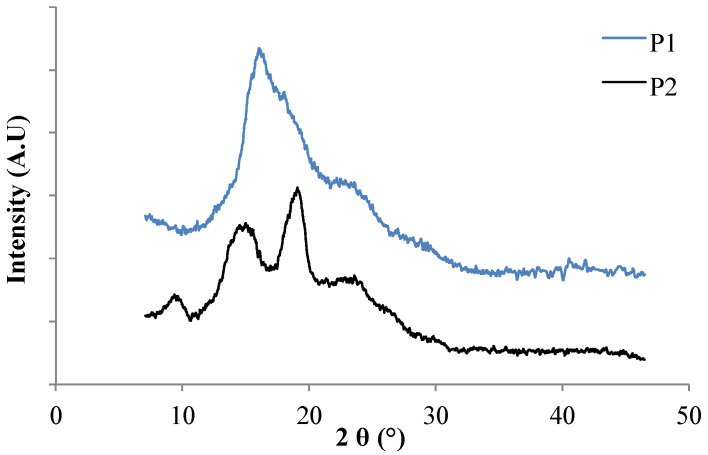
XRD diffractograms of polymers **P1** and **P2**. The two patterns are shifted along the ordinate for better clarity.

The X-ray powder diffraction (XRD) technique was used to investigate the structure of polymers **P1** and **P2**. Figure 2 shows the diffractograms obtained for the two polymers, where differences concerning both the position and the resolution of diffraction peaks can be observed. For **P1**, the broad diffraction pattern indicated that this polymer was rather amorphous. In contrast, **P2** exhibited some crystalline characteristics, with a crystallinity index in the order of 71% and a crystallite size of (4.9 ± 0.1) nm for the main peak, corresponding with 2θ at around 19° (using the Debye–Scherrer equation). The π–π stacking distance was found to be in the order of 3.1 Å, which was close to that found by Z-F Yao et al. of, typically, 3.4 to 3.6 Å for investigated conjugated polymers [35]. The apparent dissimilarity between the two polymers was mainly due to the difference in the effects of “CH_3_” and “CF_3_”, where the latter seemed to prevent inter-chain interactions, thus leading to less crystalline characteristics for **P1**. The observed disparity emphasized the solubility results, where **P1** was very soluble in DMF, unlike **P2**; this agreed with the fact that solubility generally decreases with an increase in crystallinity.

### 3.2. Thermal Properties

The stability of amorphous glassy states at/or above room temperature is necessary to assure the good performance of hole/electron transport materials [36]. DSC and TGAs, using a nitrogen atmosphere, were thus performed to examine the thermal properties of the **P1** and **P2** polymers. Before these analyses, both polymers were dried overnight at 80 °C to eliminate residual humidity, i.e., slightly adsorbed water molecules.

For both polymers, the second-run heating scan for the DSC measurements showed a comparable glass transition temperature (Tg) in the order of 188 °C. This result was similar to those obtained for azomethine naphthalenediimides with thiophene (Tg = 195 °C) and bithiophene (Tg = 174 °C) moieties [9].

**Figure 3:**
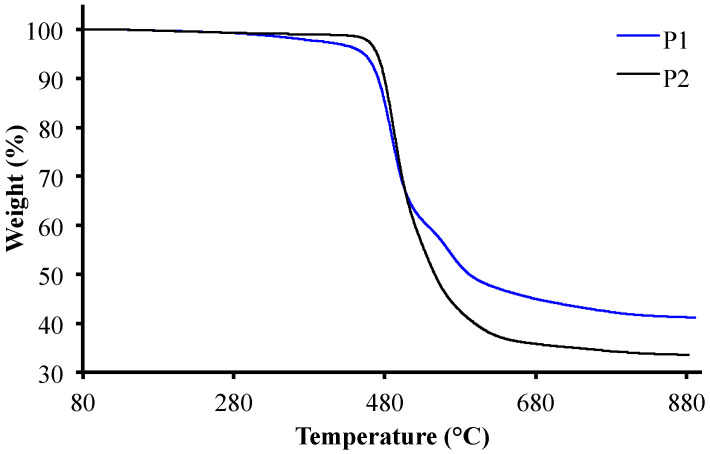
Thermogravimetric curves of polymers **P1** and **P2**.

The thermal stability of **P1** and **P2** was also investigated using thermogravimetric analyses. Variations in weight versus temperature are presented in Figure 3. Stability up to ≈ 490 °C was revealed for both polymers. The first stage of thermal degradation showed no mass changes at temperatures up to 343 °C and 439 °C for **P1** and **P2**, respectively. For **P1**, a slight decrease was observed from 343 °C, probably due to the chains’ depolymerization. The presence of a slight shoulder, more visible by decorrelation or by spectral derivatization, could indicate the presence of two different degrees of polymerization. From about 460 °C, both polymers showed the start of the degradation process, with a steep slope in mass loss. The total mass loss was also comparable in both cases (≈ 40% for **P1** and ≈ 34% for **P2**). Beyond the few differences observed, these measurements showed that it was possible to use these two polymers at temperatures up to 450 °C without any risk of thermal degradation.

The DSC and TGA analyses clearly showed that **P1** and **P2** presented high thermal stability and high glass transition temperature (Tg) values. However, the thermal properties did not seem to depend on the nature of the “R group” in the synthesized poly(azomethines) because **P1** and **P2** presented very similar thermal properties.

### 3.3. Optical Properties

**Figure 4:**
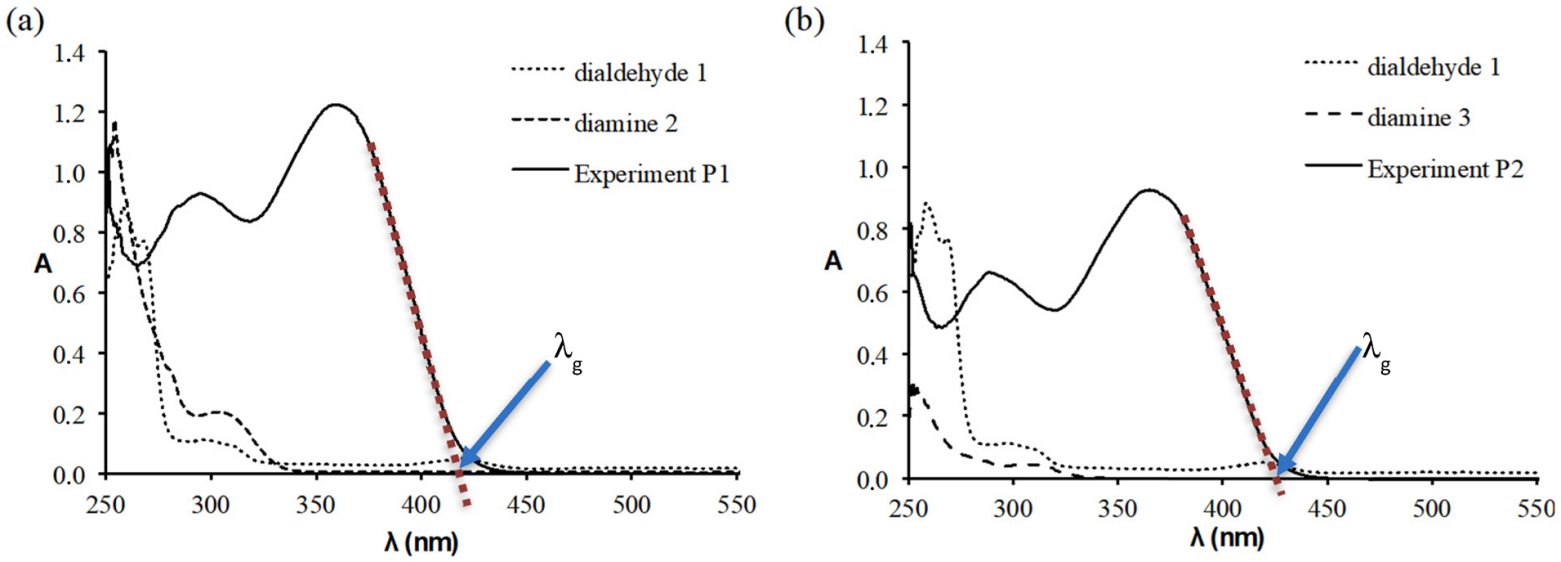
Experimental UV/Vis spectra (absorbance A versus wavelength λ) of (**a**) **P1** and (**b**) **P2**, respectively, in THF solution.

The UV/Vis spectra (recorded using THF) of the poly(azomethines) **P1** and **P2** are presented in Figure 4a,b. The results highlighted the great similarity between both polymers because the same observations were made. We noticed the presence of two absorption bands, the first in the 260–320 nm range and the second in the 320–410 nm range. The UV maximum absorption wavelength (λ_abs-max_), which was assigned to the *π*–π^*^ transition, was about 360 nm for **P1** and 367 nm for **P2**. λ_abs-max_ was slightly blue-shifted for the former, presumably due to the presence of the CF_3_-C-CF_3_ function.

Compared with the reference data for diamine **3** and dialdehyde **1**, for which the absorption maximums in THF were localized at 304 nm and 298 nm, respectively, the present poly(azomethine) exhibited significantly red-shifted *π*–π^*^ maximal absorption. This was not surprising because it is well known that an extension of conjugation contributes to a bathochromic shift as well as a hyperchromic effect in the UV/Visible absorption maximum [37]. In our systems, electron delocalization was extended on the whole chromophoric group, including aryl-amine and aryl-aldehyde patterns via the imine linkage into the polymer chain.

Using a linear approximation of the absorption edge, we could estimate the limit wavelength *λ_g_* (*λ_g_* was estimated by asymptotically extending the absorption curve to the point of intersection with the wavelength axis, as shown in Figure 4) at which radiation was no longer absorbed as well as its corresponding energy *E_g_* using Equation (1) [37].

(1) $$E_{g}=\frac{hc}{\lambda_{g}}$$

where *h* is the Planck constant and *c* is the velocity of light.

The values of *λ*_abs-max_, *λ_g_*, and *E_g_* estimated from the spectra are gathered in Table 3 for both polymers. There was no significant difference between the **P1** and **P2** polymers in terms of the values of the three parameters considered. These highlighted properties make the present poly(azomethines) attractive for applications in advanced opto-electronics and other related fields.

### 3.4. Conductivity

We subsequently studied the macroscopic electrical behavior of the two oligomers **P1** and **P2** by measuring their thin-film σ conductivity. The σ values for the undoped samples were in the order of 1 × 10^−8^ S/cm for **P1** and 3 × 10^−9^ S/cm for **P2** (Table 4). These values made them rather semi-insulating like most intrinsic organic polymers. Therefore, we began doping them with various dopants, including hydrochloric acid (HCl), diiodine (I_2_), paratoluenesulfonic acid (p-TsOH), and camphosulfonic acid (ACS). This doping process introduced defects that could act as extrinsic charge carriers.

To record the conductivity values of the various doped polymers, we shaped them according to their degree of solubility into thin films by spin-coating or thin disks by pressing. The results are shown in Table 4. The lack of results for doping with I_2_ was due to the difficulty in shaping the samples to obtain a film with a continuum of material to enable correct conductivity measurements to be obtained.

It should be noted that attempts to dope the **P2** polymer with paratoluenesulfonic acid resulted in an insoluble gum, making it impossible to shape it into a pressed pellet or spin-coat as a thin film and thus to determine its conductivity. For the **P1** polymer, the conductivity was enhanced as its value increased by three orders of magnitude. Doping with ACS produced no noticeable effect.

**Figure 5:**
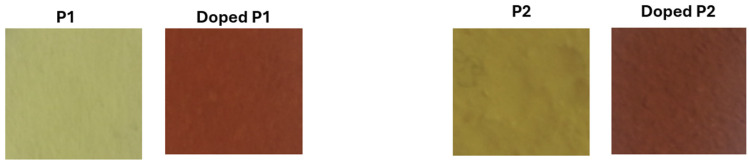
Photos of polymers **P1** and **P2** before and after doping with HCl.

The best doping was obtained with HCl, as can qualitatively be seen from the photos of the samples before and after doping (Figure 5), which showed a clear change in color. Quantitatively, the conductivity values showed a large increase of almost five orders of magnitude for both polymers, reaching values of 1.2 × 10^−3^–2.6 × 10^−3^ S·cm^−1^. To enable a rational judgement on these conductivity values, we placed them in relation to other polyazomethines. E. Sonker et al. showed that their synthesized polymers were thermally stable up to 270 °C and presented low band gaps associated with conductivity in the order of 10^−5^ S·cm^−1^ [38]. The investigations of A. Hafeez et al. presented the electrical conductivity of polyazomethine polymers in the range of 0.019–0.051 mS·cm^−1^ [39]. A. G. El-Shekeil et al. used iodine and concentrated sulfuric acid as acceptor dopants to increase polymer conductivity from the dielectric domain (10^−11^–10^−10^ S·cm^−1^) to the semiconductor domain (10^−7^–10^−6^ S·cm^−1^) [40]. L. Xiaochang et al. showed that doping conjugated polymers with iodine produced an increase in conductivity of seven to nine orders of magnitude, reaching 10^−4^ to 10^−3^ S·cm^−1^ [41].

Accordingly, we could classify our doped synthesized polymers as good semiconductors, opening the possibility of various applications. The gap between the values achieved using other reference polymers such as PEDOT was still significant, but this approach could prove to be very promising [42].

## 4. Towards Semiconducting Organic Materials

As part of the growing effort to develop organic electronic devices, we considered cellulose filaments, an environmentally friendly, biodegradable product from renewable resources, as an interesting substrate for the deposition of the doped **P1** and **P2** polymers.

### 4.1. Cellulose Filaments as a Support

In addition to their renewable and biodegradable nature, the choice of cellulose filaments was motivated by their high strength, flexibility, lightness, and, above all, their very specific bonding properties, which make them exceptional reinforcement products. The aim of this part of the study was to pave the way for the development of semiconductor paper devices based on the principle of the physical adhesion of polymers to the surface of cellulose filaments.

The cellulose filaments were obtained after the mechanical treatment of softwood pulp fibers. Each consisted of approximately 1000 cellulose filaments with a length of between 500 and 1000 µm, a thickness of 40 to 100 nm, and a width of 80 to 300 nm.

**Figure 6:**
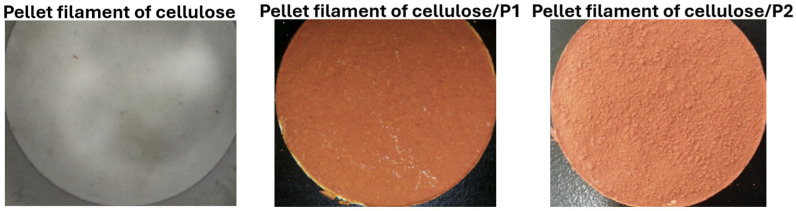
Photos of the cellulose-filament thin disks before and after deposition of the **P1** and **P2** semiconducting polymers.

Semiconductor deposition was carried out in thin-disk form, as shown in Figure 6. After several trials, optimum deposits were obtained using a 1:1 mass ratio of cellulose filaments to doped polymers. To achieve this, cellulose-filament thin disks were pre-pressed at 5,000 kPa for 30 s. The polymers in fine powder form were then dispersed on the surface of these disks, and the whole product was put under pressure at 10,000 kPa for 5 min.

Once deposited, separating the polymers from the cellulose filaments’ surface was impossible without destroying the pellet’s integrity. This showed the existence of strong physical adhesion between both products.

**Figure 7:**
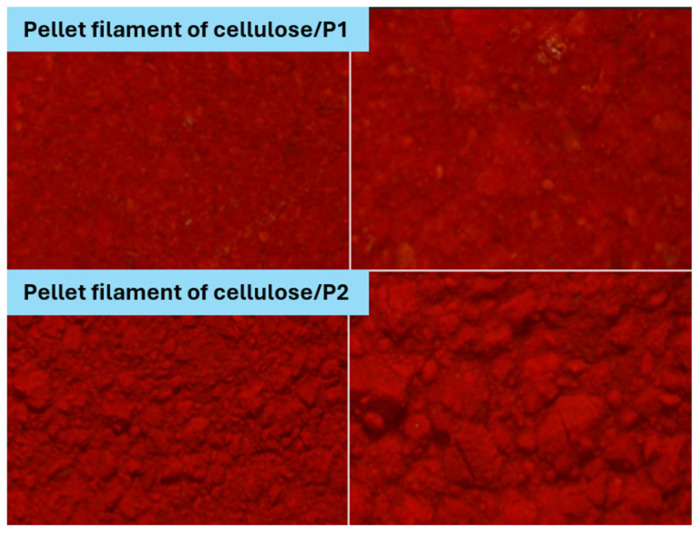
Stereoscope images of filament/polymer surfaces at 20X (**left**) and 50X (**right**) magnification.

A stereoscope was used to check the homogeneity and relief of the deposits. As can be seen in Figure 7, the **P1**- and **P2**-doped polymers homogeneously covered the entire surface of the cellulose filaments. In the case of the filament/**P2** pellet, we observed roughness due to aggregate formation. Despite this, adhesion between the cellulose filaments and the polymer remained very strong.

An AFM was used to probe the resulting materials’ morphology and ensure their surface homogeneity and continuity at the nanoscale. The measurements were conducted in phase contrast mode to verify the absence of discontinuities and decorrelate the effects of relief and topography from those due to possible chemical variations [43].

**Figure 8:**
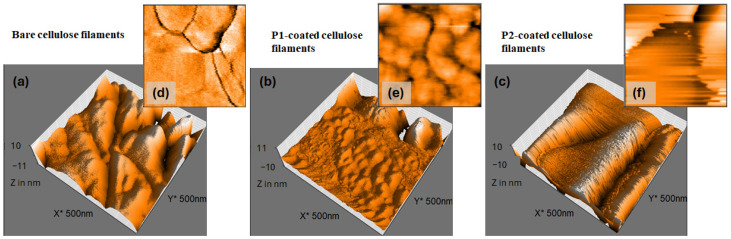
AFM 3D topographic images (**a**–**c**) for an area of (0.5 × 0.5) μm^2^ and 2D phase contrast images (**d**–**f**) over an area (200 × 200) nm^2^.

The results obtained are shown in Figure 8, which shows the 3D topographic images (Figure 8a–c) for an area of (0.5 × 0.5) μm^2^ and the 2D phase contrast images (Figure 8d–f) over a smaller area (200 × 200) nm^2^ to better highlight the differences.

These images revealed that the asperities representing the surface of the pressed cellulose filaments varied in size from 70 nm to 180 nm on average. For the cell/**P1** sample, the sizes were smaller and mostly varied between 30 nm and 40 nm. The cell/**P2** surface showed larger asperities (120 nm to 300 nm), again in relation to the size of the **P2** particles. In terms of roughness values, whose main parameters (arithmetic average, root mean square, and peak-to-valley height) are gathered in Table 5, the cellulose filaments presented a rougher surface than **P1**- and **P2**-coated cellulose. The latter presented a particular shape that probably corresponded with a grain joint, explaining the very high peak-to-valley height value.

### 4.2. Determination of Conductivity

Once it had been ensured that the deposits resulted in resistant and sufficiently homogeneous materials, the conductivity of this new material was checked and compared with that of the base polymers. The measurements were obtained using the same equipment and procedure as for the **P1** and **P2** polymers, and the results obtained are shown in Table 6.

The results showed no difference in conductivity, within uncertainties, between the doped polymers before and after deposition on cellulose filaments. This indicated that the deposition method preserved the semiconducting properties of the polymers, paving the way for producing planar semiconducting structures on sheet-like cellulose filaments using the stamping method.

### 4.3. Diffuse Reflectance Spectroscopy

**Figure 9:**
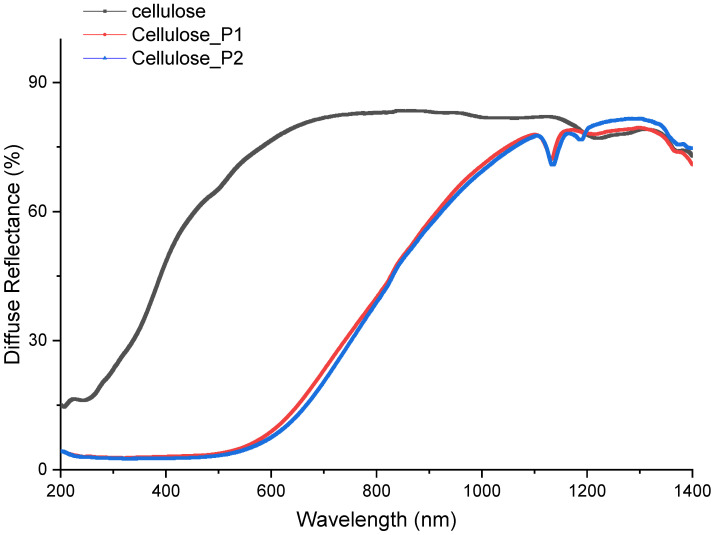
Diffuse reflectance spectra of cellulose-filament sample and polymer-coated cellulose filaments versus wavelength.

The diffuse reflectance spectra (DRS) of the cellulose-filament sample and those of the **P1**- and **P2**-polymer-coated cellulose filaments were recorded in the wavelength range of 200–1400 nm (Figure 9). Compared with bare cellulose filaments, the polymer coating resulted in a noticeable red shift, with a relative similarity between the two polymers regarding radiation absorption.

DRS measurements were obtained to determine the band gap energy of the studied samples. The most common approach relies on the Kubelka–Munk (K-M) method. The K-M function, denoted as *F*(*R*), was estimated from diffuse reflectance measurements according to Equation (2) [44,45].

(2) $$F(R)=\frac{{\left(1-R\right)}^{2}}{2\;R}$$

where *F*(*R*) is the Kubelka–Munk (K-M) function and *R* is the reflectance.

Then, a relationship could be assumed between the modified K-M function and the optical band gap *E_g_* (Equation (3)).

(3) $${\left(F(R)\times h\nu \right)}^{n}=C\times \left(h\nu -E_{g}\right)$$

where *C* is an empirical constant, *hν* is the photon energy, *E_g_* is the optical gap, and *n* is an exponent whose value depends on the nature of optical transitions where *n* = 2 for an allowed direct transition and *n* = 1/2 for an allowed indirect one [45].

From here, and by giving the value of ½ or 2 to the exponent *n*, the plot of (*F*(*R*) *× hν*)*^n^* as a function of *hν* could then be used to extract the bandgap value from an extrapolation of the linear part near the band edge.

**Figure 10:**
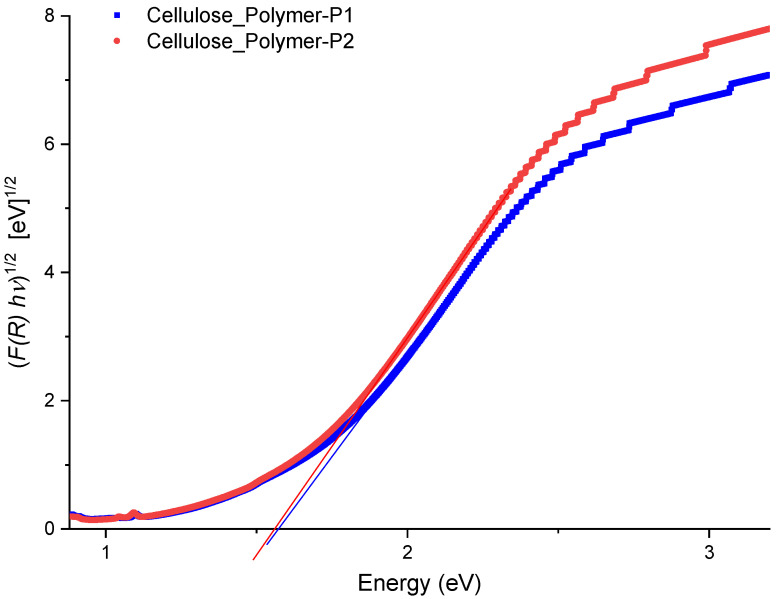
Modified Kubelka–Munk plot: (*F*(*R*) × *hν*)^1/2^ (indirect transition) as a function of energy *hν* (in eV) for the two polymer-coated cellulose-filament samples.

Numerous investigations have delved into diverse approaches to estimate the band gap energy (*E_g_*) through graphical representations, underscoring the variability in equations. It is worth highlighting that most authors do not specify the nature of the associated transitions in graphical representations. In a previous study, we presented a novel empirical approach to determine the nature of the transition before estimating the gap [46]. In the present case, we found that the transition was allowed to be indirect, which permitted us to plot (*F*(*R*) *× hν*)^1/2^ as a function of *hν* to extract the value of *E_g_* (Figure 10).

The experimental values of the optical gap (*E_g_*) obtained by extrapolation of the linear parts were (1.57 ± 0.15) eV and (1.56 ± 0.15) eV for **P1**- and **P2**-coated cellulose filaments, respectively. These values were largely in the semiconductor range.

## 5. Conclusions

We successfully prepared a new poly(azomethine), **P1**, with improved solubility from the polycondensation of terephthalaldehyde and 4-BDAF under green chemistry conditions. This new hexafluoroisopropylidene-containing polymer was compared with an isopropylidenediphenyl-containing polymer, **P2**, to study the influence of fluorine atoms on the polymer properties. Both **P1** and **P2** were easily soluble in most polar aprotic solvents and even in less polar ones, which are convenient and easily accessible. However, the X-ray diffraction (XRD) showed that polymer **P2** exhibited some crystalline characteristics compared with polymer **P1**, which presented a rather amorphous structure. It appears that the presence of fluorine atoms seemed to prevent inter-chain interactions, thus leading to less crystalline characteristics. In contrast, the thermal properties were found to be not dependent on the nature of the “R group” in the synthesized poly(azomethines). Both these new polymers (**P1** and **P2**) exhibited high resistance against thermal decomposition up to 490 °C, which renders advantages in a whole variety of applications. Moreover, a large interval between the Tg (188 °C) and the decomposition temperature of the obtained poly(imines) could be advantageous in processing using the thermoforming technique. We doped **P1** and **P2** with various dopants, and the conductivity values obtained showed a significant increase of almost five orders of magnitude in the case of doping with HCl.

We used cellulose filaments, a biodegradable product derived from renewable resources, to deposit the doped **P1** and **P2** polymers. The results showed that there was no difference in the conductivity of the doped polymers before and after deposition on the filaments. This indicated that the deposition method preserved the semiconducting properties of the polymers, paving the way for producing planar semiconducting structures on sheet-like cellulose filaments using the stamping method. This heralds a host of possible applications, including sensors for health and/or environment.

## Figures and Tables

**Table 1:** Average molecular weights of the polymers.

Polymer	Mw ^a^ (g/mol)	Mn ^b^ (g/mol)	Mw/Mn (PDI) ^c^	DP ^d^
**P1**	4903	3184	1.54	8
**P2**	2324	1979	1.17	5

^a^ Weight-average molecular weight; ^b^ number-average molecular weight; ^c^ polydispersity index; ^d^ number-average degree of polymerization.

**Table 2:** Solubility behavior of the polymers ^a^.

Polymer	CHCl_3_	THF	DMF	DMAc	NMP
**P1**	++	++	++	++	++
**P2**	++	++	--	++	++

^a^ The solubility was tested by the dissolution of 1 mg of the solid polymer in 1 ml organic solvent at room temperature. (++): Soluble; (--): not soluble.

**Table 3:** Representative parameters of polymers **P1** and **P2** estimated using UV/Vis absorption spectra.

	UV/Vis Extracted Parameters		
	*λ_abs-max_* (nm)	*λ_g_* (nm)	*E_g_* (eV)
**P1**	360	420	2.95
**P2**	367	425	2.92

**Table 4:** Conductivity measurement of undoped and doped **P1** and **P2** polymers.

	Conductivity	(S/cm)
Doping Agent	P1	P2
Without doping	1 × 10^−8^	3 × 10^−9^
HCl	2.6 × 10^−3^	1.2 × 10^−3^
p-TsOH	3.2 × 10^−5^	-
ACS	2 × 10^−8^	4 × 10^−9^

**Table 5:** Roughness parameter values for bare cellulose filaments and cellulose filaments coated with **P1** and **P2** polymers.

	Roughness Parameters		
	Arithmetic Average S_a_ (nm)	Root Mean Square S_q_ (nm)	Peak-to-Valley Height S_z_ (nm)
Cell filaments	(6.1 ± 0.2)	(8.0 ± 0.3)	(86.1 ± 1.3) (nm)
**P1**-coated cell filaments	(2.6 ± 0.2)	(4.6 ± 0.3)	(44.6 ± 1.1) (nm)
**P2**-coated cell filaments	(2.8 ± 0.2)	(4.2 ± 0.2)	(1.01 ± 0.09) (µm)

**Table 6:** Conductivity measurements of doped **P1** and **P2** polymers and of polymer-coated cellulose filaments.

	Conductivity	(S/cm)
Polymer	Doped Polymer	Polymer-Coated Cellulose Filaments
**P1**	2.6 × 10^−3^	2.4 × 10^−3^
**P2**	1.2 × 10^−3^	1.3 × 10^−3^

## Data Availability

The original contributions presented in this study are included in the article. Further inquiries can be directed to the corresponding author.
